# Pharmacological assessment of *Coffea arabica* compounds as potential therapeutics for cervical cancer

**DOI:** 10.1093/bioadv/vbaf132

**Published:** 2025-06-05

**Authors:** Victor Omoboyede, Nwachukwu Christiana Okonkwo, Jimoh Olayemi Balogun, Onyekachi Victor Onyedikachi, Rita Ononiwu, Daniel Okpaise, Sarah Olanrewaju Oladejo, Christopher Busayo Olowosoke, Haruna Isiyaku Umar, Prosper Obed Chukwuemeka

**Affiliations:** Department of Biochemistry, School of Life Sciences (SLS), Federal University of Technology Akure, Akure, P.M.B 704, Nigeria; Computer-Aided Therapeutic Discovery and Design Group, Akure, Ondo State, P.M.B. 704, Nigeria; Department of Pharmaceutical and Medicinal Chemistry, Nnamdi Azikiwe University, Awka, Anambra State, P.M.B 5025, Nigeria; Department of Microbiology, Lead City University, Ibadan, Oyo State, 200255, Nigeria; Department of Microbiology, College of Natural Sciences, Michael Okpara University of Agriculture Umudike, Umuahia, Abia State, P.M.B 7267, Nigeria; Department of Chemical Sciences, Bells University of Technology, Ota, Ogun State, P.M.B 1015, Nigeria; Department of Virology, College of Medicine, University of Ibadan, Ibadan, P.M.B 3017, Nigeria; Department of Microbiology, Lagos State University, Ojo (Main campus), Lagos State, P.M.B. 0001, Nigeria; Computer-Aided Therapeutic Discovery and Design Group, Akure, Ondo State, P.M.B. 704, Nigeria; Department of Biotechnology, School of Life Sciences (SLS), Federal University of Technology Akure, Akure, P.M.B 704, Nigeria; Department of Biochemistry, School of Life Sciences (SLS), Federal University of Technology Akure, Akure, P.M.B 704, Nigeria; Computer-Aided Therapeutic Discovery and Design Group, Akure, Ondo State, P.M.B. 704, Nigeria; Computer-Aided Therapeutic Discovery and Design Group, Akure, Ondo State, P.M.B. 704, Nigeria; Department of Biotechnology, School of Life Sciences (SLS), Federal University of Technology Akure, Akure, P.M.B 704, Nigeria

## Abstract

**Motivation:**

Cervical cancer remains a leading cause of gynecological mortality, with existing treatments often limited by resistance and suboptimal efficacy. While *Coffea arabica* is rich in phytochemicals with reported anticancer properties, their relevance to cervical cancer-specific molecular targets remains underexplored. Here, we integrated transcriptomic profiling, cheminformatics, and survival modeling to evaluate the therapeutic potential of *C. arabica* compounds in cervical cancer.

**Results:**

From 158 bioactive compounds with favorable pharmacokinetic and drug-likeness properties, we predicted gene targets and intersected them with 1779 differentially expressed genes identified from bulk RNA-sequencing of 304 cervical cancer tumors and 47 normal cervical tissues. This yielded 43 *C. arabica* gene targets that were significantly dysregulated in cervical cancer. Pathway enrichment revealed involvement in tumorigenesis, immune modulation, and cell cycle regulation, with fold enrichment computed as the ratio of observed-to-expected gene overlap. Survival analysis identified 14 of these genes as markers of poor prognosis, with matrix metalloproteinase-7 (MMP7) emerging as an independent prognostic marker of adverse outcome. A Random-Forest-Regression model trained on 499 experimentally validated MMP7 inhibitors identified carnosol—a *C. arabica* compound—as a top-ranking candidate with high predicted activity. These findings nominate carnosol as a promising therapeutic lead for cervical cancer and lay the groundwork for future experimental validation.

**Availability and implementation:**

The data supporting the findings of this study, including bulk RNA-seq gene expression data, survival, and phenotype data, are available through the TCGA database. These data can be accessed via the Xenabrowser platform (https://xenabrowser.net) using the reference identifier [TCGA Cervical Cancer (CESC)]. Corresponding healthy cervical tissue RNA-seq data, are available through the Genotype-Tissue Expression (GTEx) project (https://www.gtexportal.org/home/). The codes used for differential gene expression (DGE) analysis, pathway enrichment, and survival analysis, as well as scripts for generating volcano plots (DGE analysis), Kaplan-Meier survival plots, and boxplots (gene expression), and machine learning implementations are available on GitHub (https://github.com/Ponaskillzyy/Coffea_arabica_Potential_in_Cervical_Cancer).

## 1 Introduction

With the increasing availability of data on global mortality, cancer as an expanding threat is the second leading non-communicable disease, resulting in death globally, next to cardiovascular complications (Arbyn *et al.* 2020). In this context, cervical cancer (CC) is a major cause of gynecological-related deaths worldwide (Arbyn *et al.* 2020). Despite the tireless efforts of researchers and unabated technological advancement to unravel novel approaches to elevate CC therapy in health care services, CC is still a mystery. With the remission of CC in patients, the priority on conventional-based approaches for treatment (including surgery, radiotherapy, and chemotherapy) is eroding, thus indicating that the search for new interventional therapies continues incessantly. Studies have shown that the key pathological mechanisms for CC can be attributed to the dysregulation of several oncogenes and tumor suppressor proteins and their associated signaling pathways (Manzo-Merino *et al.* 2014, Zhang *et al.* 2015). The growing understanding regarding these pathologic mechanisms of CC birth forth more innovative therapeutic options, including targeted therapy, and advancing the design and development of pre-existing therapies, such as chemotherapy and cancer immunotherapy, among which are the most broadly employed CC therapies in recent times (Menderes *et al.* 2016, Kumar *et al.* 2018). Nonetheless, the high-cost implication of molecular-targeted therapy and the negative side effects of chemotherapy are overwhelming and may result in the abandonment of treatment. Due to these concerns, substantial attention has been given to the establishment and utilization of medicinal botanicals as an important resource for discovering anticancer drug agents due to their safe pharmacological profile.

Coffee (*Coffea arabica* L), belonging to the *Rubiaceae* family, has long been used as one part of complementary medicine in various regions of the world and is endemic to the southwestern highlands of Ethiopia (Geneti 2019). Coffee leaves and beans, as the major therapeutic part of the plant, are rich in diverse groups of phytochemicals, leading to a broad spectrum of pharmacological effects (Patay *et al.* 2016). Noteworthy, the derivatives of Methylxanthine (caffeine and theophylline), the representative components derived from *C. arabica*, have been reported to induce apoptosis and autophagy in gastric cancer cells by activation of phosphatase and tensin homolog (PTEN) and inhibition of PI3K/Akt/mTOR pathway (Liu *et al.* 2019). More also, Feng *et al.* (2005) revealed that chlorogenic acids from coffee bean extract belonging to the group caffeoylquinic acids (CQAs) protect against environmental carcinogen-induced carcinogenesis. They further emphasized that the observed chemoprotective activities of CQAs were attributable to the suppression of AP-1, ROS-mediated NF-kB, and MAPK activation, all of which are located downstream of the small GTPase Ras. Noteworthy, these pathways play crucial roles in cell proliferation and survival, as their activation is commonly associated with promoting cancer cell growth and resistance to apoptosis. Interestingly, Maugeri *et al.* (2018) also reported that caffeine significantly declines the expression levels of HIF-1α and VEGF in glioblastoma cells exposed to hypoxia, through the inhibition of MAPK/ERK and PI3K/Akt signaling pathways (Maugeri *et al.* 2018). In addition, studies on Caspase-3 activation in CRL5985 and HTB182 lung cancer cell lines revealed that treatment with caffeine augments the apoptotic actions of cisplatin on the cells restraining cell proliferation (Wang *et al.* 2015).

**Figure 1.**
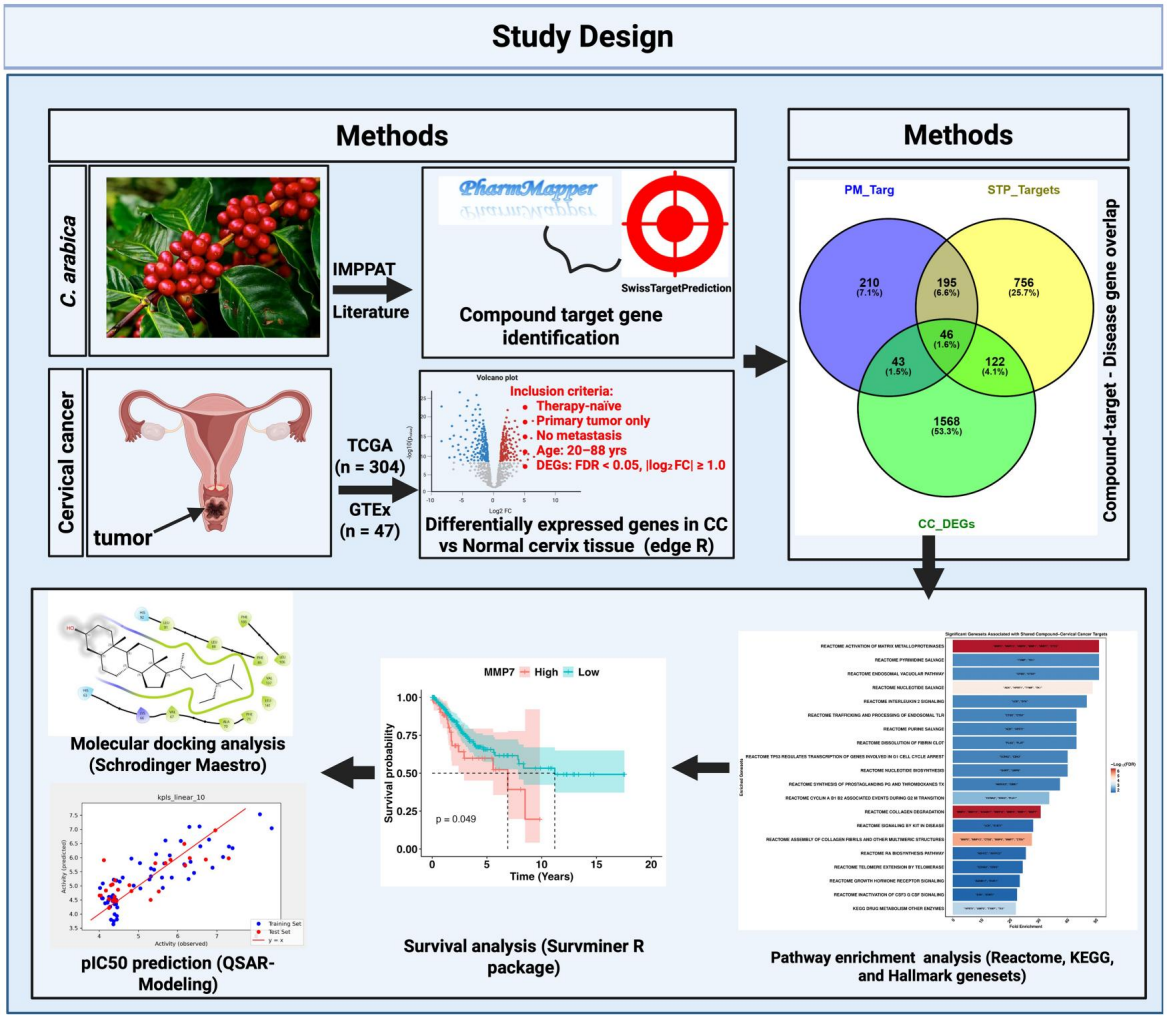
Schematic diagram illustrating analysis workflow.

While previous studies have highlighted the broad anticancer potential of bioactive compounds in *C. arabica*, including their ability to induce apoptosis and interfere with oncogenic signaling in various malignancies, there remains limited understanding of how these compounds might interact with gene targets relevant to CC. Given the complex molecular landscape of CC, characterized by dysregulated oncogenes and tumor suppressor pathways, a rational approach to identifying actionable targets is essential for natural product-based drug discovery. To address this, our study integrates transcriptomic analysis, absorption-distribution-metabolism-excretion-toxicity (ADMET) screening, survival-based gene prioritization, and molecular modeling to systematically evaluate the pharmacological potential of *C. arabica* compounds in CC. By combining these complementary analyses, we aim to identify clinically relevant gene targets and predict their interactions with *C. arabica*-derived phytochemicals, providing a data-driven foundation for future natural product-based therapeutic strategies in CC. The overall workflow of this study is presented in Fig. 1.

## 2 Methods

### 2.1 Data acquisition and preprocessing

#### 2.1.1 Construction of the chemical library

The therapeutic components of *C. arabica* were obtained from the Indian Medicinal Plants, Phytochemistry and Therapeutics curated database (IMPPAT) (https://cb.imsc.res.in/imppat/home) which is a distinctive Indian herbal medicines pharmacology platform that captures phytochemical composition, traditional medicinal formulation, and disease interactions (Mohanraj *et al.* 2018). In addition to this database, we consulted two previously published studies by Ali *et al.* (2022) and Zhao *et al.* (2024), which identified bioactive compounds present in *C. arabica*. Then, the candidate compounds were screened using pharmacokinetic (PK) models in drug absorption, distribution, metabolism, and excretion process, including drug-likeness using the Lipinski rule of 5 (RO5) (Chen *et al.* 2020).

#### 2.1.2 Acquisition of compound targets

Potential targets of *C. arabica* active ingredients were obtained through online target prediction platforms, PharmMapper (http://lilab-ecust.cn/pharmmapper/), and the SwissTarget Prediction (http://www.swisstargetprediction.ch/) (Liu *et al.* 2010, Daina *et al.* 2019).

#### 2.1.3 Acquisition of gene expression datasets

Primary tumor gene expression data for CC patients (*n* = 304) were obtained from The Cancer Genome Atlas (TCGA) CESC cohort. Inclusion criteria encompassed therapy-naive patients with primary tumors and no metastatic disease, aged between 20 and 88 years. Corresponding healthy cervical tissue RNA-seq data (*n* = 47) were sourced from the Genotype-Tissue Expression (GTEx) project. All datasets were processed uniformly to ensure compatibility for downstream analyses.

### 2.2 Differential gene expression analysis

Differential expression analysis between primary tumors and normal tissues was conducted using the edgeR (v4.0.16) R package (Robinson *et al.* 2010). Genes that did not meet a minimum count per million (CPM) thresholds of 100 in at least 2 samples were excluded to ensure data quality, and genes lacking current annotation were removed from the analysis. Library compositional differences were normalized using the trimmed mean of M-values (TMM) method (Robinson and Oshlack 2010). The counts were transformed to log_2_-CPM for downstream analysis. To identify differentially expressed genes (DEGs), a design matrix was created to model the sample groups, and pair-wise comparisons were specified using custom contrasts. Dispersion estimates were obtained using quantile-adjusted conditional maximum likelihood estimation. A negative binomial generalized linear model was fitted to the data using the quasi-likelihood (QL) approach (glmQLFit), providing robust control over type I error rates. Differential expression was assessed through QL *F*-tests (glmQLFTest), which offer greater precision in variance estimation compared to other methods. The *P*-values obtained from the QL model were adjusted for multiple testing using the Benjamini–Hochberg method to control the false discovery rate (FDR) at 5%.

### 2.3 Mapping compound targets to CC-relevant genes

The predicted targets of the filtered *C. arabica* compounds were collected and overlapped with the DEGs identified from the TCGA versus GTEx analysis using the Venny 2.1 webserver (https://bioinfogp.cnb.csic.es/tools/venny/) (Oliveros 2007). Genes that were both differentially expressed and targeted by the compounds were considered disease-relevant and selected for further investigation. A total of 46 shared genes were initially identified from this intersection. However, following validation via the Wilcoxon rank-sum test, only 43 genes demonstrated consistent differential expression and were retained for subsequent analyses.

### 2.4 Gene set acquisition and functional enrichment analysis

To gain insights into the biological functions and signaling pathways associated with the 43 validated shared genes, we conducted pathway over-representation analysis (ORA) prior to clinical relevance filtering. Gene sets were retrieved from the MSigDB database using the msigdbr R package (Cillo *et al.* 2024), focusing on the C2 Reactome subset (1615 gene sets), Kyoto encyclopedia of genes and genomes (KEGG) canonical pathways (186 gene sets), and the Hallmark gene set collection (50 gene sets). Pathway enrichment was performed using the enricher function from the ClusterProfiler R Bioconductor package. Gene sets with an adjusted *P*-value < 0.05 after FDR correction were considered significantly enriched. Fold enrichment was calculated, and results were visualized using custom R scripts (available at: https://github.com/Ponaskillzyy/Coffea_arabica_Cervical_cancer_mechanism). Noteworthy, the fold enrichment is a metric used in ORA to quantify how much a particular gene set (e.g. a biological pathway) is overrepresented in a subset of genes of interest (such as DEGs) compared to what would be expected by chance.


(1) $$\mathrm{Fold}\;\mathrm{Enrichment}\;=\frac{\begin{array}{c} (\mathrm{Number}\;\mathrm{of}\;\mathrm{genes}\;\mathrm{in}\;\mathrm{the}\;\mathrm{gene}\;\mathrm{set})/\\ \left(\mathrm{Total}\;\mathrm{number}\;\mathrm{of}\;\mathrm{genes}\;\mathrm{in}\;\mathrm{the}\;\mathrm{background}\right) \end{array}}{\begin{array}{c} (\mathrm{Number}\;\mathrm{of}\;\mathrm{genes}\;\mathrm{in}\;\mathrm{the}\;\mathrm{gene}\;\mathrm{set})/\\ \left(\mathrm{Total}\;\mathrm{number}\;\mathrm{of}\;\mathrm{genes}\;\mathrm{in}\;\mathrm{the}\;\mathrm{background}\right) \end{array}}$$

### 2.5 Prioritization of clinically relevant genes

#### 2.5.1 Univariate survival analysis

To assess the clinical significance of the 43 overlapping genes, univariate survival analyses were performed. Bulk RNA-seq expression data and corresponding clinical information from CC patients in the TCGA CESC cohort were accessed via the Xenobrowser platform. Only primary tumor samples used in the DEG analysis with available survival data were included. Raw RNA-seq counts were normalized using the log-CPM method in R Bioconductor package edgeR. Patients were stratified into high- and low-expression groups based on optimal cut-points identified using the surv_cutpoint function from the survminer R package. Kaplan–Meier survival analysis was then conducted to evaluate the association of each gene’s expression with overall survival.

#### 2.5.2 Multivariate Cox-proportional hazards model

Genes that demonstrated a significant association with poor survival in univariate analysis were further analyzed using multivariate Cox-proportional hazards models to identify those independently correlated with patient outcomes. Multivariate analyses were performed using the survival (v3.2-11), survminer (v0.4.9), and survMisc (v0.5.5) R packages. Variables were considered independently associated with survival if their hazard ratios remained significant after adjustment for confounders. This process led to the prioritization of four genes for further investigation. To validate the robustness of our model, we included LCK and STAT1 as internal controls. Both genes have been previously associated with improved patient outcomes in CC. Notably, high STAT1 expression has been linked to enhanced radiosensitivity and favorable prognosis in CC patients. The consistent protective effect observed for LCK and STAT1 in our analyses supports the reliability of our multivariate model.

### 2.6 Quantitative structure–activity relationship model development for bioactivity prediction

To predict the potential bioactivity of *C. arabica* compounds against matrix metalloproteinase-7 (MMP7), a quantitative structure–activity relationship (QSAR) model was developed. A total of 625 experimentally tested MMP7 inhibitors were initially retrieved from the BindingDB database (https://www.bindingdb.org/rwd/bind/index.jsp). Compounds lacking specific IC_50_ values were excluded, resulting in a curated dataset of 499 inhibitors for model development.

#### 2.6.1 Data transformation

Given the wide range of IC_50_ values and to reduce anharmonicity, IC_50_ values were transformed to pIC_50_ values using the formula:


(2) $$p{\mathrm{IC}}_{50}={-\log}_{10}({\mathrm{IC}}_{50}[M])$$

This transformation standardizes the data, facilitating more effective modeling and interpretation.

#### 2.6.2 Descriptor calculation and preprocessing

Molecular descriptors, encompassing one-dimensional (1D), two-dimensional (2D), and three-dimensional (3D) features, were computed using the PaDEL-Descriptor software via the PaDelpy package. An initial set of 1875 descriptors was generated. Descriptors with near-zero variance were removed to eliminate non-informative features, resulting in 873 retained descriptors. Further feature selection was performed using a recursive feature elimination (RFE) strategy to identify the most predictive subset of descriptors. This process yielded 20 informative descriptors.

To ensure the independence of these descriptors, pairwise correlation analysis was conducted. The analysis confirmed that the selected descriptors were non-orthogonal, thereby minimizing redundancy and enhancing the predictive power of the model.

#### 2.6.3 Model training and validation

A random forest (RF) regression model was trained using the caret R package. The dataset was randomly split into a training set (80%) and a test set (20%). Model performance was evaluated based on standard metrics including R^2^, root mean square error (RMSE), and mean absolute error. To ensure robustness, a 10-fold cross-validation procedure was implemented during model training.

#### 2.6.4 Bioactivity prediction of C. arabica compounds

The trained QSAR model was subsequently used to predict the pIC_50_ values of the 165 *C. arabica* compounds. Predicted bioactivities were compared to known MMP inhibitors such as Barimastat and the cognate ligand of MMP7. The compound carnosol was identified as having superior predicted activity relative to these controls.

### 2.7 Molecular docking simulation

The crystal structure of MMP7 was obtained from the protein data bank (https://www.rcsb.org/, PDB ID: 2Y6C) and was prepared using the Protein Preparation Workflow available in Schrodinger Maestro, and it was executed as reported by Ojedele *et al.* (2024). Subsequently, the 3D structure of *C. arabica* active compounds was obtained from the PubChem database (https://pubchem.ncbi.nlm.nih.gov). The retrieved compounds were subjected to preparation using the LigPrep module of Schrodinger Maestro, after which they were docked against the active site of the protein identified based on the position of the cognate ligand using the Extra Precision algorithm of the Glide tool.

### 2.8 Quantification and statistical analyses

All analyses were performed using R version 4.3.2 (2023-10-31). Specifically, the survival package (v3.2-11) and survminer (v0.4.9) were utilized to generate prognostic survival curves. To independently validate the differential gene expression results obtained from the edgeR QL framework, a non-parametric two-sided Wilcoxon rank-sum test was performed. This test was applied to compare gene expression distributions between tumor and normal tissues using the stat_compare_means function from the ggpubr R package. This orthogonal assessment served to reinforce the robustness of the identified DEGs, particularly by confirming statistical significance in the absence of distributional assumptions.

## 3 Results

### 3.1 *C. arabica* contains compounds with drug-like properties and desirable PK profiles

**Figure 2.**
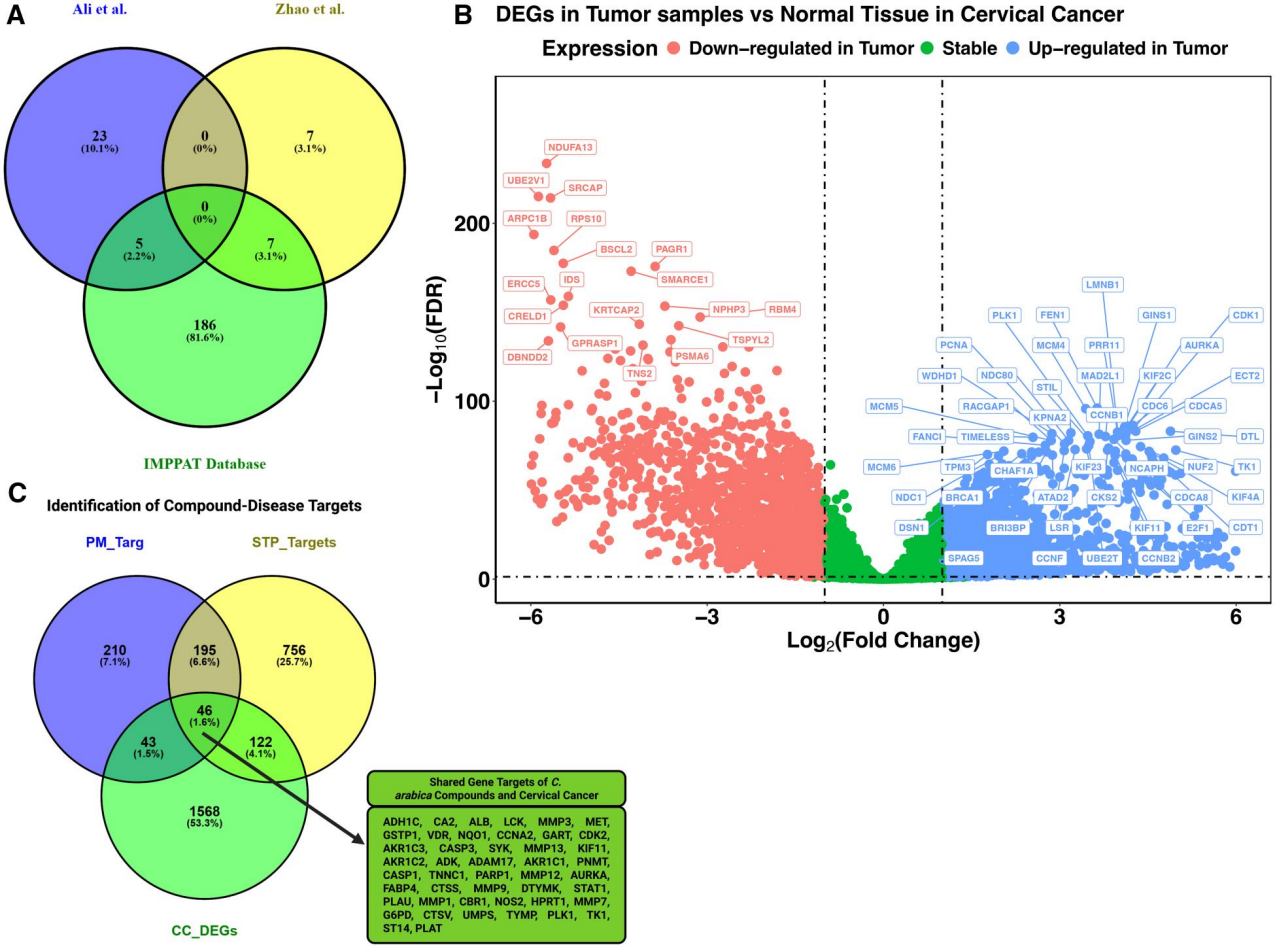
Identification of candidate therapeutic targets of *Coffea arabica* compounds in cervical cancer. (A) Venn diagram illustrating the overlap between bioactive compounds retrieved from the IMPPAT database and those curated from published literature, representing the final pool of unique *C. arabica* phytochemicals. (B) Volcano plot displaying the DEGs identified from transcriptomic analysis of cervical cancer tumors (TCGA-CESC, *n* = 304) versus normal cervical tissues (GTEx, *n* = 47). Significantly upregulated and downregulated genes are highlighted based on FDR < 0.05 and |log_2_FC| > 1. (C) Venn diagram showing the intersection between predicted targets of *C. arabica* compounds and DEGs in cervical cancer, identifying shared genes that are both dysregulated in the disease and potentially modulated by the phytochemicals.

In this study, we employed an integrated computational pipeline combining transcriptomic analysis, ADMET-based compound screening, survival-guided gene prioritization, machine learning-driven bioactivity prediction, and molecular docking simulations to assess the pharmacological potential of *C. arabica* compounds in CC. Our investigation commenced with a comprehensive screening of *C. arabica* bioactive compounds using the IMPPAT phytochemical database and supporting literature. A total of 198 unique compounds were retrieved from the IMPPAT database, while 28 and 14 compounds were identified from studies by Ali *et al.* (2022) and Zhao *et al.* (2024), respectively (Table 1, available as supplementary data at *Bioinformatics Advances* online). Interestingly, five compounds from Ali *et al.* and seven from Zhao *et al.* overlapped with those retrieved from the IMPPAT database (Fig. 2A). These compounds span major phytochemical classes, including terpenes and terpenoids, phenolic compounds, alkaloids, and fatty acids/lipids.

Given that systemic toxicity and adverse side effects have led to the discontinuation of many small-molecule drugs in clinical use, we prioritized evaluating the PK and toxicity profiles of the identified *C. arabica* compounds. Our analysis revealed 201 compounds exhibited favorable PK and safety profiles, suggesting their therapeutic potential. The compounds with undesirable properties are colored red in Table 1, available as supplementary data at *Bioinformatics Advances* online. Accumulating literature highlights that many of the bioactive components possess anticancer properties, exemplified by Caffeine and Theophylline, which have been reported to induce apoptosis and autophagy in gastric cancer cells by activation of PTEN and inhibition of PI3K/Akt/mTOR pathway (Liu *et al.* 2019).

### 3.2 Overlap between *C. arabica* compound targets and upregulated genes in CC identifies genes potentially mediating therapeutic effects

Recognizing that the therapeutic effects of small-molecule drugs are mediated through specific interactions with molecular targets that initiate cascades of cellular and biochemical responses, we sought to identify potential gene targets of *C. arabica* compounds within the human biological system. Consequently, we performed target prediction using the PharmMapper and SwissTargetPrediction web servers, leading to the identification of 1372 unique gene targets. Specifically, 494 and 1119 targets were identified via PharmMapper and SwissTargetPrediction, respectively, with an overlap of 231 targets between the two platforms. Noteworthy, due to limitations in PharmMapper’s predictive coverage, only 158 compounds from *C. arabica* yielded identifiable gene targets. These compounds, each supported by target predictions from at least one validated source, were prioritized for downstream functional and pathway analyses to enhance biological relevance and interpretative coherence.

The identified gene targets included members of various families such as estrogen receptors, cyclin-dependent kinases, MMPs, ribonucleases, glutathione S-transferases, heat shock proteins, cytochrome P450s, and aldo-keto reductases, among others.

Since the predicted targets of *C. arabica* compounds are broad and may not be specific to CC, we performed differential gene expression analysis using TCGA RNA sequencing data. This helped us narrow down the list to only those genes that are relevant to CC, ensuring a more focused identification of potential therapeutic targets.

**Figure 3.**
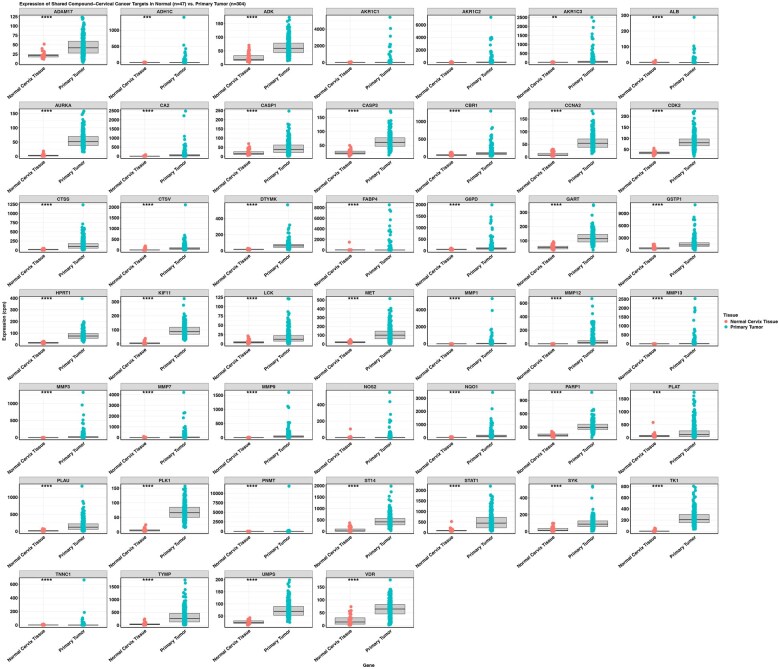
Boxplots illustrating the expression profiles of the 46 shared target genes. Each boxplot displays the differential expression of genes shared between *Coffea arabica* compound targets and cervical cancer DEGs, comparing healthy cervical tissue (GTEx, *n* = 47) and cervical cancer tumor samples (TCGA-CESC, *n* = 304). Note, expression levels are shown per patient to capture inter-individual variability. Wilcoxon rank-sum test was used for statistical analysis. *P*-values: *P* < 0.05; *P ≤ 0.05, **P ≤ 0.01, ***P ≤ 0.001, ****P ≤ 0.0001.

To identify disease-relevant gene targets, we analyzed bulk RNA sequencing data from TCGA to identify DEGs by comparing primary tumor samples from CC patients (n = 304) against healthy cervical tissues obtained from the GTEx database (*n* = 47). This analysis revealed 1779 DEGs, providing a robust dataset for subsequent target identification (Fig. 2B and File 1, available as supplementary data at *Bioinformatics Advances* online). We then overlapped the predicted targets of *C. arabica* compounds with the DEGs to identify genes potentially mediating therapeutic effects. This intersection initially identified 46 shared genes (Fig. 2C). However, after validating these genes using the Wilcoxon rank-sum test, only 43 demonstrated consistent differential expression, with AKR1C1, AKR1C2, and NOS2 excluded. These 43 genes were retained for further analyses (Fig. 3).

### 3.3 Functional enrichment of genes shared between compound targets and CC DEGs reveals associations with key cancer pathways

**Figure 4.**
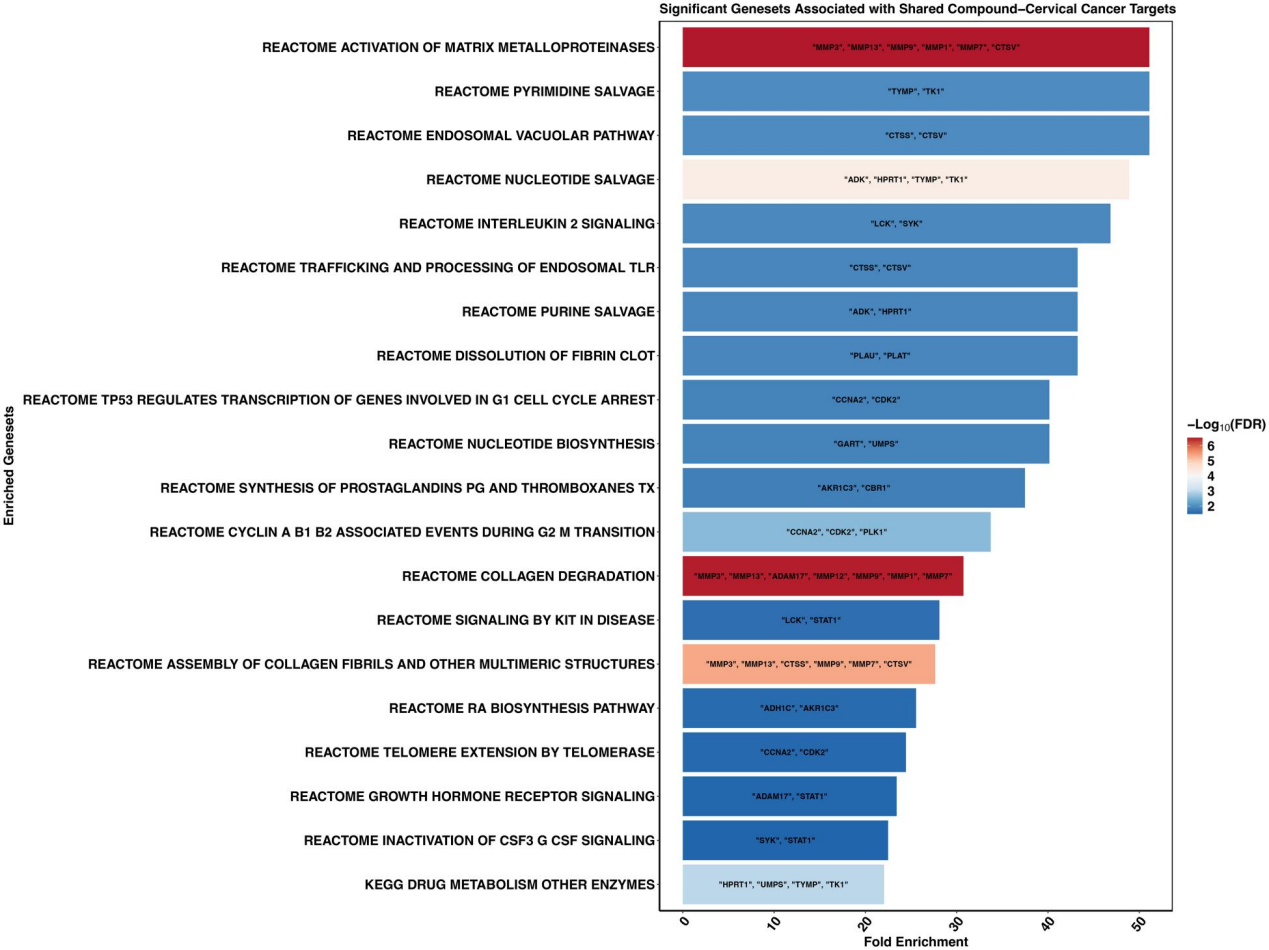
Top 20 enriched pathways derived from the 43 shared target genes. Pathway over-representation analysis was performed using curated gene sets from MSigDB, including Reactome, KEGG, and Hallmark collections. The top 20 pathways were ranked by statistical significance (adjusted *P*-value), reflecting biological processes potentially modulated by *Coffea arabica* compounds in cervical cancer.

To better understand the biological relevance of these 43 shared genes, we first performed pathway ORA using a curated list of gene sets from the MSigDB database, including the C2 Reactome subset (1615 gene sets), KEGG canonical pathways (186 gene sets), and the Hallmark gene set collection (50 gene sets). This initial analysis allowed us to explore the broader biological processes and signaling pathways potentially influenced by *C. arabica* compound targets in the context of CC. The results revealed that the shared genes were significantly enriched in 54 gene sets, including Reactome’s degradation of the extracellular matrix, hallmark KRAS (Kirsten rat sarcoma viral oncogene homolog) signaling, and KEGG pathways in cancer, as well as several pathways related to tumorigenesis, metastasis, immune modulation, and cell cycle regulation (File 2, available as supplementary data at *Bioinformatics Advances* online). The top 20 enriched pathways are illustrated in Fig. 4. Notably, several of these pathways are central to cancer progression. For example, the REACTOME pathways for activation of MMPs and collagen degradation, in which several MMPs are enriched, play a crucial role in the remodeling of the extracellular matrix (ECM), an essential process for tumor invasion and metastasis (Mustafa *et al.* 2022).

Additionally, the identification of pathways such as TP53 regulates transcription of genes involved in G1 cell cycle arrest and cyclin A/B1/B2-associated events during G2/M transition highlights the disruption of key cell cycle checkpoints commonly observed in cancerous tissues. The TP53 pathway, by inducing CDKN1A (p21), inhibits cyclin-dependent kinases, thereby promoting G1 arrest. Similarly, the regulation of cyclins A and B1/B2 is crucial for the G2/M transition, and their dysregulation can lead to unchecked cell proliferation (Balasubramaniam *et al.* 2019). Beyond classical oncogenic pathways, several enriched gene sets underscore the dysregulation of immune and endocrine signaling pathways. These include interleukin-2 signaling, trafficking and processing of endosomal toll-like receptors, and growth hormone receptor signaling. Notably, Interleukin 2 (IL-2) signaling has been implicated in CC progression, with research suggesting that IL-2 not only promotes tumor cell proliferation but also modulates the immune microenvironment, contributing to tumor growth and immune evasion (Gutiérrez-Hoya *et al.* 2024).

Additionally, the enrichment of purine and pyrimidine salvage pathways, along with nucleotide biosynthesis processes, reflects the heightened metabolic demands of rapidly proliferating CC cells. These pathways are frequently upregulated in response to oncogenic drivers such as HPV E6 and E7, which enhance nucleotide synthesis by activating MYC and disrupting tumor suppressor functions (Gore *et al.* 2025). Recent studies by Zhang *et al.* and Jia *et al.* have identified purine metabolism as a central pathway associated with CC progression. Metabolomic analyses from these studies revealed elevated levels of glycine and other amino acids critical for nucleotide biosynthesis, underscoring the metabolic reprogramming characteristic of proliferative tumor cells (Jia *et al.* 2023, Zhao *et al.* 2023). Although these studies do not explicitly highlight the salvage pathway, the enrichment of salvage enzymes such as thymidine kinase 1 (TK1)—a key player in this pathway—suggests its potential involvement. TK1 activity is often elevated in proliferating cells and has been associated with tumor progression in various cancers (Bitter *et al.* 2020). Moreover, the enrichment of pathways such as REACTOME activation of MMPs, collagen degradation, and dissolution of fibrin clot underscores the involvement of ECM remodeling processes, which are central to tumor invasion and metastasis—highlighting the clinical relevance of these molecular events in CC progression.

### 3.4 Overexpression of MMP7 and three other shared targets independently correlates with poor prognosis in CC patients

**Figure 5.**
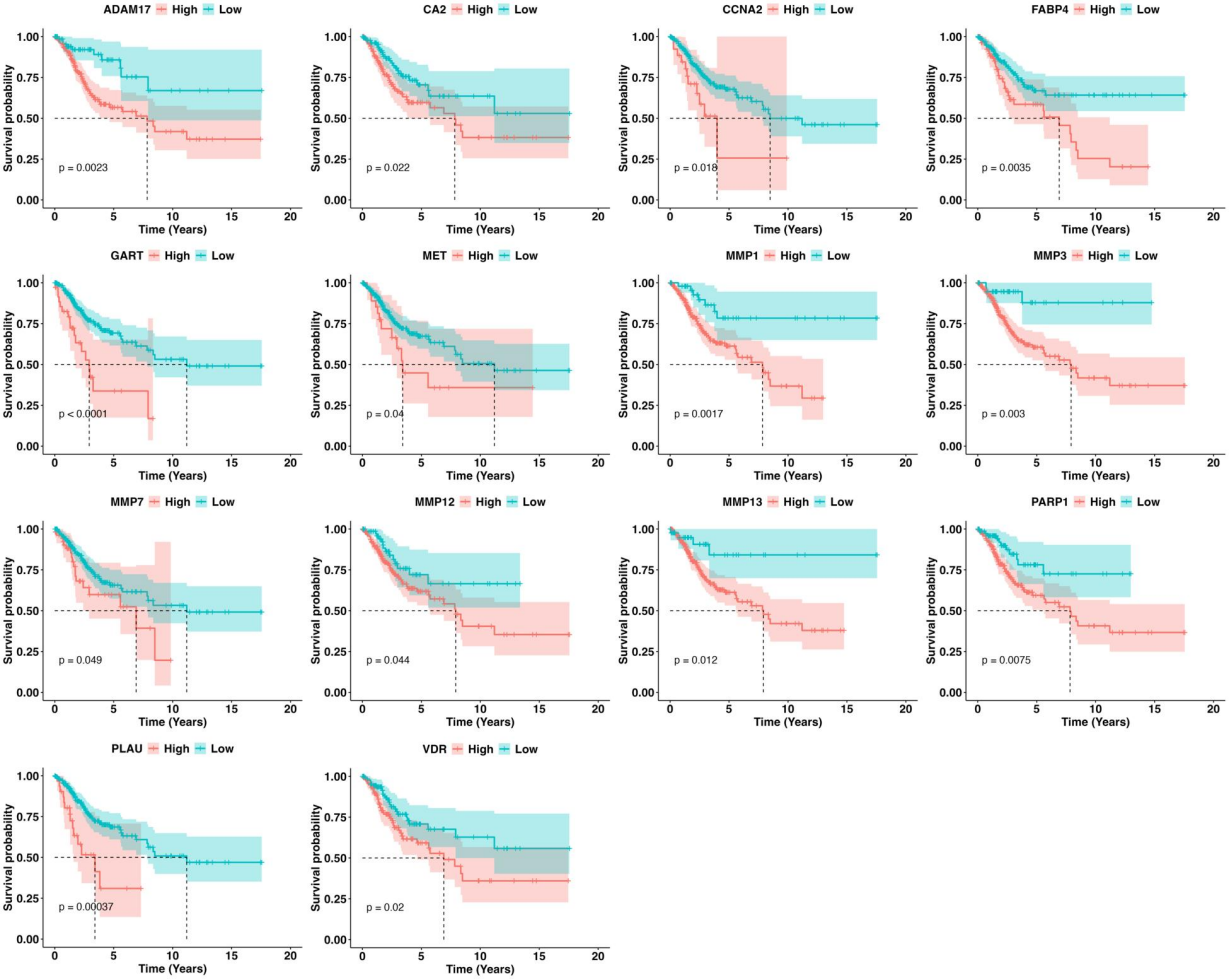
Univariate Kaplan–Meier survival plots for fourteen shared genes associated with poor prognosis in cervical cancer. Using data from the TCGA-CESC cohort, we performed univariate survival analysis by stratifying patients into high and low expression groups for each of the 43 shared genes based on median expression levels. Fourteen genes were found to significantly correlate with reduced overall survival (log-rank test, *P* < 0.05), highlighting their potential prognostic relevance in cervical cancer.

While the 43 shared genes are collectively associated with key cancer-related pathways, not all may contribute equally to disease progression. Some genes may play pivotal roles in driving poor clinical outcomes, while others may have limited or neutral impact. Therefore, to efficiently prioritize therapeutic targets, it is crucial to distinguish the key molecular drivers of CC from those with less prognostic significance. Consequently, we reasoned that prioritizing these genes based on their clinical relevance would be essential. To do so, we performed Kaplan–Meier survival analysis, correlating the expression of each of the 43 shared genes with overall survival in CC patients using data from the TCGA dataset. Our analysis identified 14 genes, including CCNA2, ADAM17, FABP4, MMP1, MMP3, and PARP1, that were significantly associated with poor prognosis in CC patients (Fig. 5). Interestingly, no significant correlation was observed for other genes, such as CASP1, CASP3, AURKA, G6PD, and MMP9, among others, which is noteworthy given their known involvement in various cancer-related pathways. The lack of a significant association with overall survival suggests that these genes may not be as critical in CC progression, or their role in disease may be context-dependent (Fig. 1, available as supplementary data at *Bioinformatics Advances* online).

**Figure 6.**
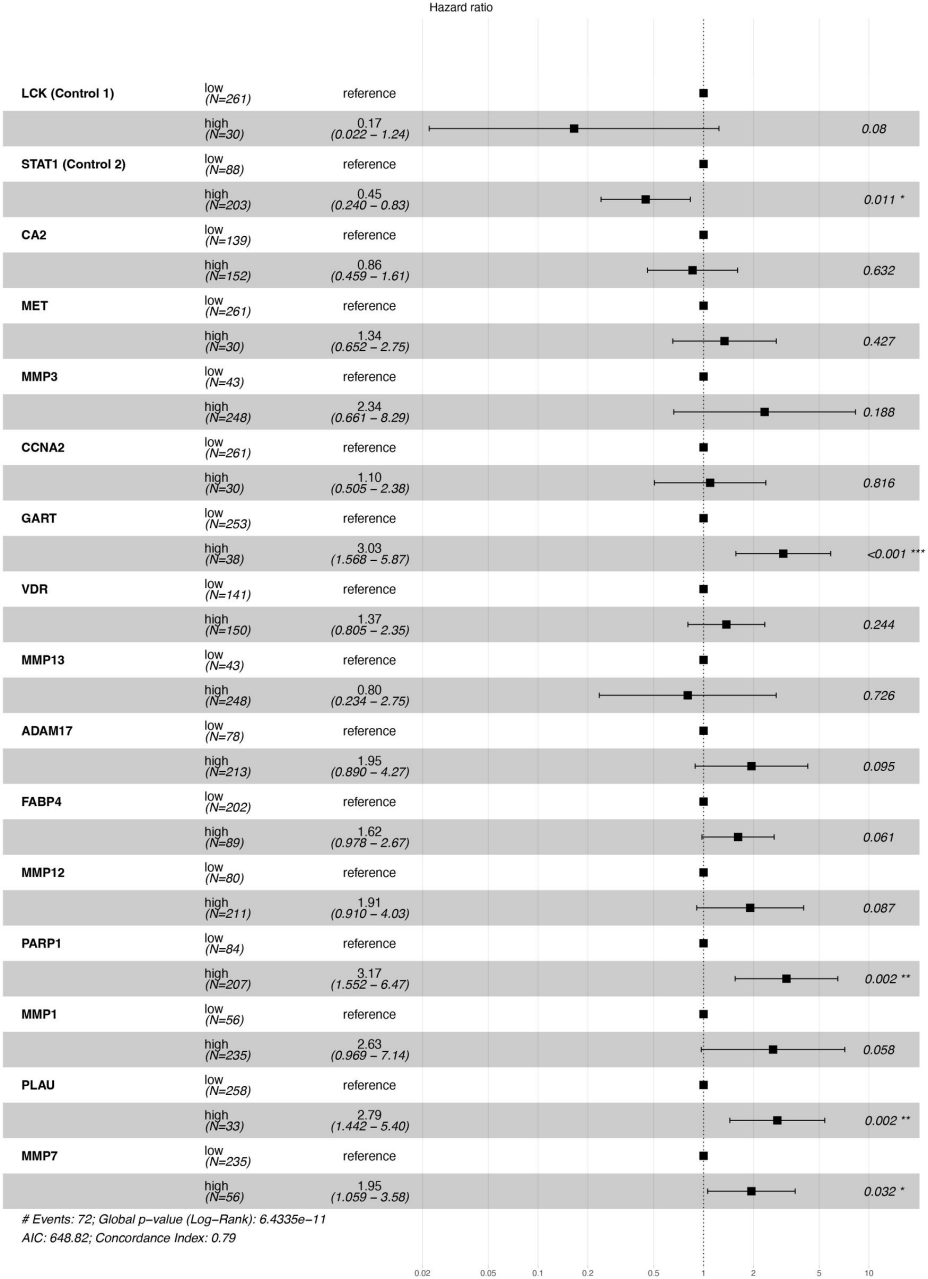
Multivariate Cox proportional hazard regression analysis of 14 genes associated with poor prognosis in cervical cancer. This plot shows the results of the multivariate Cox proportional hazards regression analysis, identifying genes that independently and significantly correlate with poor prognosis among the 14 shared genes identified in the univariate analysis. The hazard ratios (HR) for each gene are displayed along with 95% confidence intervals. Genes with HR values >1.0 are associated with an increased risk of poor survival, while HR values <1.0 indicate a protective effect. Genes with *P* < 0.05 are highlighted, suggesting their potential as independent prognostic markers for cervical cancer survival.

While 14 genes were identified as significantly associated with poor prognosis, they still represent a broad target space, making it challenging to prioritize for efficient therapeutic intervention. To refine our focus and improve target identification, we performed multivariate Cox proportional hazards regression analysis to identify genes that were independently associated with poor prognosis. This approach allowed us to account for potential interactions among variables and prioritize genes with robust, standalone prognostic significance. As shown in Fig. 6, the model demonstrated strong predictive performance, with a concordance index of 0.79 and a highly significant global log-rank P-value of 6.43 × 10^−11^.

To validate the accuracy and robustness of the model, we included LCK and STAT1 as internal controls, given that their expression does not correlate with poor prognosis. Previous studies have shown that high STAT1 expression is associated with improved survival and a protective role in cancer progression, which aligned with our findings (Wang *et al.* 2023). On the other hand, LCK showed no significant association with prognosis, consistent with its context-dependent effects. Through multivariate analysis, we narrowed down the candidate genes to four key targets—GART, PARP1, PLAU, and MMP7—which exhibited high expression levels significantly and independently associated with poorer survival outcomes. In contrast, other genes such as CA2, MET, MMP3, CCNA2, VDR, MMP13, ADAM17, FABP4, MMP12, and MMP1 did not show significant independent correlations with prognosis.

To further narrow down the key genes, we revisited the pathway analysis (Fig. 4), focusing on the distribution of the four independently prognostic genes within the top 20 enriched pathways. Notably, MMP7 appeared most frequently, underscoring its potential central role in CC biology.

### 3.5 Carnosol emerged as the most promising C. arabica-derived MMP7 inhibitor, exhibiting the highest predicted pIC_50_ and favorable binding affinity among the screened compounds

To prioritize the most promising therapeutic target for CC treatment, we focused on MMP7 among the four key genes identified in our survival analysis. While all four targets showed strong prognostic relevance, MMP7 stood out due to its recurrent involvement in several critical cancer pathways and its significant independent correlation with poor prognosis in CC patients. Given its potential central role in disease progression, MMP7 became the focal point of our subsequent bioactivity prediction and docking studies.

As part of our ongoing search for potential MMP7 inhibitors, we are specifically focused on evaluating the bioactivity of compounds derived from *C. arabica*, a plant renowned for its broad range of bioactive properties. Our goal is to explore how these compounds interact with MMP7, with an emphasis on understanding their binding affinity and potential inhibitory effects through computational analysis. By examining these molecular interactions, we aim to uncover novel therapeutic strategies that could target MMP7 and, ultimately, enhance treatment outcomes for CC.

To evaluate the bioactivity of *C. arabica*-derived compounds against MMP7, we developed a 10-fold cross-validation RF regression model using the caret package in R. A total of 625 experimentally validated MMP7 inhibitors were retrieved from BindingDB (File 3, available as supplementary data at *Bioinformatics Advances* online). After removing compounds with undefined IC_50_ values, 499 compounds were retained for model training. Using the PaDEL Python package, we extracted 1D, 2D, and 3D molecular descriptors, generating 1875 features per compound (File 4, available as supplementary data at *Bioinformatics Advances* online). To enhance model performance and eliminate redundancy, we first removed descriptors with near-zero variance, yielding 873 descriptors. We then applied RFE to identify the most informative subset of features, narrowing the list to 20 predictive descriptors.

To further assess the quality of this refined feature set, we performed a correlation analysis on the 20 selected descriptors. The goal of this analysis was to evaluate the degree of orthogonality—i.e. how independent the descriptors are from one another. Reduced orthogonality, indicated by strong correlations among descriptors, implies redundancy and could lead to multicollinearity, where the model overweights similar signals. This may decrease model generalizability and obscure the true contribution of individual descriptors.

**Figure 7.**
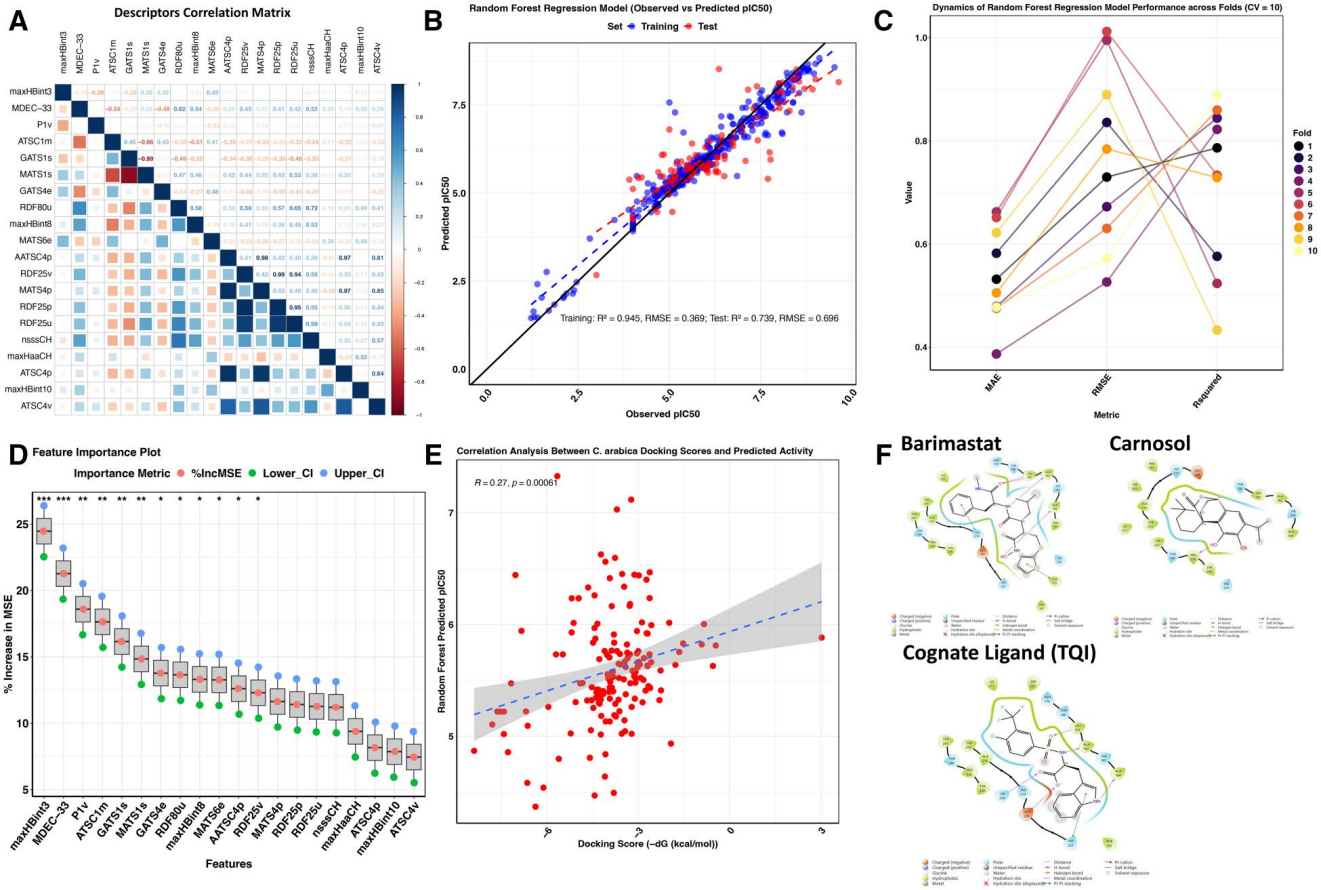
Validation of QSAR model and analysis of activity and binding interactions of *Coffea arabica* compounds against MMP7. (A) Correlation matrix of the descriptors used in the random forest regression model, illustrating the relationships between the molecular features of the compounds. (B) Scatter plot comparing the predicted pIC_50_ values to the observed pIC_50_ values for the compounds, demonstrating the predictive accuracy of the QSAR model. (C) Plot showing the dynamics of the model’s cross-validation performance, including R^2^, MAE, and RMSE, highlighting the model's accuracy and robustness. (D) Feature importance plot, showcasing the most influential descriptors in predicting the inhibitory potency (pIC_50_) of the compounds in the model. (E) Correlation plot between the docking scores and pIC_50_ values, providing insight into the relationship between the docking affinity and the predicted inhibitory activity. (F) Interaction profiles of Batimastat, TQI (cognate ligand), and Carnosol with MMP7, demonstrating the binding interactions of these compounds at the molecular level and their potential inhibitory mechanisms.

The correlation matrix (Fig. 7A) confirmed that the final descriptor set exhibited low inter-feature correlation, indicating that the features contributed distinct and complementary information. This reduction in redundancy enhances the robustness and interpretability of the model and supports more reliable predictions of compound bioactivity against MMP7.

The RF model was trained using an 80/20 train-test split and further validated through 10-fold cross-validation. Its performance—measured by R^2^ and RMSE on both training and test sets—is summarized in Fig. 7B, while cross-validation metrics are shown in Fig. 7C. The 20 most informative molecular descriptors contributing to model performance, selected via RFE, are highlighted in the feature importance plot (Fig. 7D).

Application of our model revealed that several *C. arabica* compounds possess high predicted inhibitory activity against MMP7, with pIC_50_ values ranging from 4.37 to 7.33. Among them, carnosol emerged as the most potent, with a predicted pIC_50_ of 7.33—exceeding that of Batimastat, a well-characterized broad-spectrum MMP inhibitor (pIC_50_ = 7.12), and the cognate ligand TQI bound to the selected MMP7 crystal structure (pIC_50_ = 5.47). Other compounds, such as carnosic acid, also exhibited strong predicted activity, with a pIC_50_ of 7.03. Notably, higher pIC_50_ values indicate greater predicted inhibitory potency.

Following bioactivity prediction, these compounds were docked against MMP7 to estimate their binding affinities and analyze their interaction profiles. To assess the consistency between predicted potency and docking performance, we computed the Pearson correlation between pIC_50_ values and docking scores. A modest positive correlation (*R* = 0.27) was observed (Fig. 7E), suggesting partial alignment between predicted bioactivity and binding affinity. Complete docking results and pIC_50_ predictions are available in Files 5 and 6, available as supplementary data at *Bioinformatics Advances* online, respectively. Representative interactions of carnosol, Batimastat, and cognate ligand with MMP7 are illustrated in Fig. 7F.

## 4 Discussion

In this study, we applied an integrative approach combining transcriptomic analysis, machine learning-based bioactivity prediction, and molecular docking to evaluate the therapeutic potential of *C. arabica* compounds in CC. While prior studies have explored the anticancer properties of *C. arabica* in other cancer models, this is, to the best of our knowledge, the first to systematically investigate its therapeutic relevance in CC, leveraging genomic insights alongside predictive modeling and *in silico* screening techniques.

One of the key findings of this study is the identification of four prognostic biomarkers—GART, PARP1, PLAU, and MMP7—whose overexpression was significantly associated with poor overall survival in CC patients. Through both univariate Kaplan–Meier survival analysis and multivariate Cox regression modeling, we demonstrated that these genes are independent predictors of patient prognosis, regardless of other clinical factors. GART (phosphoribosylglycinamide formyltransferase), an enzyme involved in the de novo purine biosynthesis pathway, emerged as a potential driver of CC progression. Cancer cells often exhibit increased nucleotide synthesis via the *de novo* pathway to support their rapid proliferation (Villa *et al.* 2019). Elevated GART expression has been implicated in the growth and survival of various malignancies, including breast and colorectal cancers (Agarwal *et al.* 2020, Cipolletti *et al.* 2023). A study by Cipolletti *et al.* found that high GART expression correlates with advanced tumor grade and stage, and inhibiting GART led to reduced cell proliferation (Cipolletti *et al.* 2023).

Interestingly, patients with lower GART expression levels exhibit longer relapse-free survival, highlighting its potential as a prognostic marker. Although direct studies on GART’s role in CC are limited, its involvement in nucleotide biosynthesis and its overexpression in other cancers suggest it may contribute to CC progression. Similarly, PARP1 (poly (ADP-ribose) polymerase 1), a nuclear enzyme crucial for the base excision repair pathway, plays a key role in detecting and repairing single-strand DNA breaks (Spiegel *et al.* 2021). PARP1 has been shown to be overexpressed in CC and other malignancies, where its upregulation confers a survival advantage by enabling cells to efficiently repair DNA damage induced by oncogenic stress, chemotherapy, or radiation therapy (Raspaglio *et al.* 2021).

PLAU (urokinase-type plasminogen activator) is a serine protease that plays a pivotal role in ECM degradation, facilitating processes like cell migration and invasion by converting plasminogen into plasmin. In CC, PLAU is often overexpressed, and this upregulation has been linked to poor prognosis, enhanced metastatic potential, and tumor progression, primarily through its role in promoting epithelial-mesenchymal transition. A study by Gao *et al.* (2023) demonstrated that silencing PLAU expression in CC cell lines significantly inhibited proliferation, migration, and invasion, highlighting its crucial role in CC pathogenesis. Similarly, MMP7, also known as Matrilysin, is a zinc-dependent endopeptidase that degrades various components of the ECM and modulates non-ECM substrates like E-cadherin, Fas ligand, and pro-tumorigenic cytokines. In CC, MMP7 is frequently overexpressed, with higher levels correlating with increased tumor invasion, lymph node metastasis, and poor clinical outcomes. Silencing MMP7 in CC cell lines has been shown to decrease proliferation, migration, and invasion, further reinforcing its role in cancer progression (Zhu *et al.* 2018).

Among the four independent prognostic biomarkers identified through multivariate analysis, MMP7, a matrix metalloproteinase involved in ECM remodeling, was significantly enriched in several top-ranked pathways, including REACTOME activation of MMPs, REACTOME collagen degradation, and REACTOME assembly of collagen fibrils and other multimeric structures. These pathways are integral to ECM breakdown, turnover, and remodeling—processes that are hallmark features of cancer cell invasion and metastasis (Conlon and Murray 2019). Within the REACTOME Activation of MMPs pathway, MMP7 plays a role in the proteolytic activation of other MMPs, such as proMMP-1, proMMP-8, and proMMP-9, thereby amplifying pro-tumorigenic effects through upregulation of related biological processes (Juurikka *et al.* 2019). Additionally, MMP7’s enrichment in the collagen degradation pathway indicates its role in dismantling structural ECM components, notably type IV collagenase, as well as activating other MMPs, including MMP1 and MMP8 (Kaczorowska *et al.* 2020, Ii *et al.* 2006). The repeated presence of MMP7 across these interconnected pathways highlights its central regulatory role in ECM dynamics. In the context of CC, where enhanced ECM remodeling facilitates tumor invasion, angiogenesis, and immune evasion (Ii *et al.* 2006), MMP7’s prominent involvement in these processes, coupled with its more frequent occurrence relative to other prognostic biomarkers, provided an interesting rationale for prioritizing it as a therapeutic target. This comprehensive understanding led us to focus on evaluating bioactive compounds derived from *C. arabica* for their potential inhibitory effects on MMP7.

Another interesting finding of this study is the identification of carnosol from *C. arabica* as having favorable inhibitory potential against MMP7. Carnosol, a naturally occurring phenolic diterpene, has been extensively studied for its wide range of pharmacological properties, including antioxidant, anti-inflammatory, and anticancer activities (Kashyap *et al.* 2017). Its anticancer potential has been investigated across various cancer models, including leukemia, breast, skin, prostate, and colon cancers, among others (Johnson 2011). In colon cancer cell lines, carnosol has been shown to induce apoptosis by upregulating caspase-3, caspase-9, PARP, and p53 in a dose-dependent manner, while downregulating MDM2 (Park *et al.* 2014). Similarly, in MCF-7 cell lines, a 48-hour treatment with carnosol led to a dose-dependent decrease in cell viability and downregulation of androgen receptor and estrogen receptor-alpha expression (Johnson *et al.* 2010).

Molecular docking analysis revealed that carnosol exhibits a favorable binding interaction with MMP7. Carnosol forms a hydrogen bond with Pro239, and engages in extensive hydrophobic interactions with residues such as Leu181, Ala182, Ala184, Phe197, Trp203, Ile211, Ala216, Tyr215, Tyr238, Tyr241, and Pro239—effectively spanning the enzyme’s S1 and S1’ pockets. Additional polar interactions with Thr180, His183, His219, His229, and Thr240, and a negatively charged electrostatic interaction with Glu220, suggest a stable and well-oriented binding conformation. Our control compounds, namely Batimastat and the cognate ligand (TQI), also interacted with the same set of amino acid residues. Notably, Batimastat formed hydrogen bonds with LEU181, ALA182, and ALA184. Similarly, TQI interacted with LEU181, ALA182, ALA184, and HIS229 via hydrogen bonds. Batimastat and TQI interacted with other amino acid residues not present in the interaction of carnosol with MMP7; these include PHE103, TYR172, ASN179, and HIS223 among others. However, carnosol demonstrated a higher predicted pIC_50_ value and docking score than Batimastat. While its pIC_50_ value also exceeded that of TQI, it exhibited a comparatively lower predicted binding affinity.

These observations align with findings by Edman *et al.*, who highlighted the structural plasticity of MMP7’s S1’ pocket as a typically shallow cavity. Their crystallographic data showed that bulky ligands could induce conformational rearrangements in Tyr215 and Tyr241, enabling the pocket to expand and accommodate large side chains (Edman *et al.* 2011). Notably, carnosol interacts directly with Tyr215 and Tyr241, indicating its potential to exploit similar pocket-opening mechanisms. The involvement of Glu220 is particularly significant; in Edman *et al.*’s study, it facilitated hydrogen bonding with the carboxylate group of inhibitors, stabilizing their orientation near the catalytic zinc. Though carnosol lacks a classical zinc-chelating moiety, its engagement with Glu220 may compensate via electrostatic attraction. Additionally, Ala216, a residue shown to be critical for MMP7 selectivity due to its smaller side chain compared to other MMPs, participates in a hydrophobic contact with carnosol. This unique residue positioning, alongside carnosol’s structural complementarity, underscores its potential as a selective MMP7 modulator. Collectively, these insights suggest that carnosol may inhibit MMP7 activity through both direct interactions and induced-fit adaptation of the S1’ pocket.

In summary, our findings position carnosol, identified from several *C. arabica* compounds, as a promising candidate for inhibiting MMP7, a key mediator of ECM degradation in CC. By potentially restoring the integrity of the ECM, carnosol may help limit the invasion and metastasis of CC cells. These results offer a reasonable scientific foundation for further experimental investigation of carnosol as a therapeutic agent for the management of CC.

## Supplementary Material

vbaf132_Supplementary_Data

## Data Availability

The data supporting the findings of this study, including bulk RNA-seq gene expression data, survival, and phenotype data, are available through the TCGA database. These data can be accessed via the Xenabrowser platform (https://xenabrowser.net) using the reference identifier [TCGA Cervical Cancer (CESC)]. Corresponding healthy cervical tissue RNA-seq data, are available through the Genotype-Tissue Expression (GTEx) project (https://www.gtexportal.org/home/).

## Code availability

The codes used for differential gene expression (DGE) analysis, pathway enrichment, and survival analysis, as well as scripts for generating volcano plots (DGE analysis), heatmaps (docking scores), Kaplan-Meier survival plots, and boxplots (CCNA2 gene expression), are available on GitHub (https://github.com/Ponaskillzyy/Coffea_arabica_Potential_in_Cervical_Cancer).
