# Supplementary material for: Pharmacological assessment of Coffea arabica compounds as potential therapeutics for cervical cancer
Source: Bioinform Adv. 2025 Jun 5;5(1):vbaf132. doi: 10.1093/bioadv/vbaf132 (PMC12212767; doi:10.1093/bioadv/vbaf132)
Supplement: vbaf132_Supplementary_Data [file vbaf132_supplementary_data.zip › Supplementary_Table_1.pdf]

Supplementary Table 1. List of C. arabica bioactive compounds retrieved from the IMPPAT server and literature, and the details of their pharmacokinetics screening.

S/N	COMPOUNDS	PHYTOCHEMICAL IDENTIFIER	RO5	ABSORPTION				METABOLISM (CYP450 Inhibition)					TOXICITY		
				$Cac0_2$	BBB	HIA	P-gp	IA2	2C9	2D6	2C19	3A4	AT	HT	hERG
1.	2-Hexanone	CID:11583	+	+	+	+	-	No	No	No	No	No	No	No	No
2.	2-Hexanol	CID:12297	+	+	-	+	+	No	No	No	No	No	No	No	No
3.	Benzyl Alcohol	CID:244	+	+	-	-	-	No	No	No	No	No	No	No	No
4.	Methyl salicylate	CID:4133	+	+	-	+	+	No	No	No	No	No	Yes	No	No
5.	Myrcene	CID:31253	+	+	+	+	-	No	No	No	No	No	No	No	No
6.	Neryl formate	CID:5354882	+	+	+	+	-	No	No	No	No	No	No	No	No
7.	Geranyl formate	CID:5282109	+	+	+	+	-	No	No	No	No	No	No	No	No
8.	Cinnamyl acetate	CID:5282110	+	+	-	+	-	Yes	No	No	No	No	No	Yes	No
9.	Methyl phenylacetate	CID:7559	+	+	-	+	-	Yes	No	No	No	No	No	Yes	No
10.	2-Phenylethanol	CID:6054	+	+	-	-	-	Ye	No	No	No	No	No	No	No
11.	Ethyl benzoate	CID:7165	+	+	-	+	-	Yes	No	No	No	No	No	No	No
12.	Methyl benzoate	CID:7150	+	+	-	+	-	Yes	No	No	No	No	No	No	No
13.	Phenylacetonitrile	CID:8794	+	+	-	+	-	Yes	No	No	No	No	No	No	NO
14.	Methyl anthranilate	CID:8635	+	+	-	-	-	No	No	No	No	No	No	No	No
15.	Indole	CID:798	+	+	+	+	-	Yes	No	No	No	No	No	No	No
16.	Methyl 2-(methylamino)benzoate (Dimethyl anthranilate)	CID:6826	+	+	-	+	-	No	No	No	No	No	No	No	No
17.	6-Methyl-5-hepten-2-one	CID:9862	+	+	+	+	-	No	No	No	No	No	No	No	No
18.	Ethyl acetate	CID:8857	+	+	-	+	-	No	No	No	No	No	No	No	No
19.	Phenylacetaldehyde	CID:998	+	+	-	+	-	No	No	No	No	No	No	No	No
20.	Benzyl acetate	CID:8785	+	+	-	+	-	Yes	No	No	No	No	No	No	No
21.	Benzaldehyde	CID:240	+	+	-	+	-	Yes	No	No	No	No	No	No	No
22.	2,6-Dimethyl-5-heptenal	CID:61016	+	+	+	+	-	No	No	No	No	No	No	No	No
23.	Terpinolene	CID:11463	+	+	+	+	-	No	No	No	No	No	No	No	No
24.	Citral	CID:638011	+	+	+	+	-	No	No	No	No	No	No	No	No
25.	Linalool	CID:6549	+	+	+	+	-	No	No	No	No	No	No	No	No
26.	alpha-Terpineol	CID:17100	+	+	+	+	-	No	No	No	No	No	No	No	No
27.	Nerol	CID:643820	+	+	+	+	-	No	No	No	No	No	No	No	No
28.	(Z)-beta-Ocimene	CID:5320250	+	+	+	+	-	No	No	No	No	No	No	No	No
29.	(E)-beta-ocimene	CID:5281553	+	+	+	+	-	No	No	No	No	No	No	No	No
30.	Limonene	CID:22311	+	+	+	+	-	No	No	No	No	No	No	No	No
31.	2,3-Pentanedione	CID:11747	+	+	-	+	-	No	No	No	No	No	No	No	No
32.	1-Penten-3-Ol	CID:12020	+	+	-	+	-	No	No	No	No	No	No	No	No
33.	2-Heptanol	CID:10976	+	+	+	+	-	No	No	No	No	No	No	No	No
34.	Isovaleraldehyde (3-Methylbutanal)	CID:11552	+	+	-	+	-	No	No	No	No	No	No	No	No
35.	Xanthine	CID:1188	+	-	-	-	-	No	No	No	No	No	Yes	No	No

36.	(E)-5-(3,3-dimethyloxiran-2-yl)-3-methylpent-2-en-1-ol	CID:11126706	+	+	-	+	-	No	No	No	No	No	Yes	No	No
37.	3-Hexanol	CID:12178	+	+	-	+	-	No	No	No	No	No	No	No	No
38.	Methyl N-formylanthranilate	CID:162458	+	+	-	+	-	Yes	No	No	No	No	No	No	No
39.	Hypoxanthine	CID:790	+	+	-	-	-	No	No	No	No	No	No	No	No
40.	Caffeine	CID:2519	+	+	-	+	-	No	No	No	No	No	No	Yes	No
41.	Tetradecanal	CID:31291	+	+	+	+	-	No	No	No	No	No	No	No	No
42.	cis-2-Penten-1-ol ((Z)-2-Penten-1-ol)	CID:5364919	+	+	-	+	-	No	No	No	No	No	No	No	No
43.	gamma-Terpinene	CID:7461	+	+	+	+	-	No	No	No	No	No	No	No	No
44.	cis-Cadin-4-en-7-ol	CID:91746528	+	+	+	+	-	No	Yes	No	No	No	No	No	No
45.	L-(+)-Arabinose	CID:5460291	+	-	-	-	-	No	No	No	No	No	No	No	No
46.	D-Glucuronic Acid	CID:94715	+	-	-	-	-	No	No	No	No	No	No	No	No
47.	Safrole	CID:5144	+	+	+	+	-	No	No	No	No	No	Yes	Yes	No
48.	1-Octen-3-OL	CID:18827	+	+	+	+	-	No	No	No	No	No	No	No	No
49.	alpha-Ionone	CID:5282108	**+**	**+**	**+**	**+**	**-**	**Yes**	**No**	**No**	**No**	**No**	**Yes**	**Yes**	**No**
50.	Theophylline	CID:2153	+	-	-	+	-	No	No	No	No	No	No	Yes	No
51.	p-Cymene	CID:7463	+	+	+	+	-	Yes	No	No	No	No	No	No	No
52.	Hexanal	CID:6184	+	+	+	+	-	No	No	No	No	No	No	No	No
53.	Theobromine	CID:5429	+	-	-	+	-	No	No	No	No	No	Yes	Yes	No
54.	Benzyl salicylate	CID:8363	+	+	+	+	-	No	No	No	Yes	No	No	No	No
55.	Vanillic acid	CID:8468	+	-	-	-	-	No	No	No	No	No	No	No	No
56.	1-Hexanol	CID:8103	+	+	-	+	-	No	No	No	No	No	No	No	No
57.	Heptanal	CID:8130	+	+	+	+	-	No	No	No	No	No	No	No	No
58.	2,4-Hexadienal	CID:637564	+	+	-	+	-	No	No	No	No	No	Yes	No	No
59.	4-O-Methylglucuronic acid	CID:151010	+	-	-	-	-	No	No	No	No	No	No	No	No
60.	Paraxanthine (1,7-Dimethylxanthine)	CID:4687	+	-	-	+	-	No	No	No	No	No	No	Yes	No
61.	gamma-Curcumene	CID:12304273	+	+	+	+	-	No	No	No	No	No	No	No	No
62.	Eucalyptol	CID:2758	+	+	+	+	-	No	No	No	No	No	No	No	No
63.	4-Hydroxybenzoic acid	CID:135	+	+	-	-	-	No	No	No	No	No	No	No	No
64.	4-Carvomenthenol (4-Terpineol, (+/-)-)	CID:11230	+	+	+	+	-	No	No	No	No	No	No	No	No
65.	Chlorogenic acid	CID:1794427	+	-	-	-	-	No	No	No	No	No	No	No	No
66.	Ferulic acid	CID:445858	+	-	-	+	-	No	No	No	No	No	No	No	No
67.	(+)-gamma-Cadinene	CID:6432404	+	+	+	+	-	No	No	No	No	No	No	No	No
68.	alpha-Gurjunene	CID:15560276	+	+	+	+	-	No	Yes	No	No	No	No	No	No
69.	beta-Farnesene	CID:5281517	+	+	+	+	-	No	No	No	No	No	No	No	No
70.	alpha-Terpinene	CID:7462	+	+	+	+	-	No	No	No	No	No	No	No	No
71.	cis-3-Hexen-1-ol	CID:5281167	+	+	-	+	-	No	No	No	No	No	No	No	No
72.	2-Hexenal	CID:5281168	+	+	-	+	-	No	No	No	No	No	Yes	No	No
73.	(1R)-2-methyl-5-propan-2-ylbicyclo[3.1.0]hex-2-ene	CID:6451618	+	+	+	+	-	No	No	No	No	No	No	No	No
74.	Scopoletin	CID:5280460	+	+	-	+	-	Yes	No	No	No	No	No	No	No
75.	Phytol	CID:5280435	+	+	+	+	-	Yes	No	No	No	Yes	No	No	No
76.	alpha-Curcumene	CID:92139	+	+	+	+	-	No	No	No	No	Yes	No	No	No
77.	Sabinene	CID:18818	+	+	+	+	-	No	No	No	No	No	No	No	No

78.	alpha-Pinene	CID:6654	+	+	+	+	-	No	No	No	No	No	No	No	No
79.	Linalool	CID:6549	+	+	+	+	-	No	No	No	No	No	No	No	No
80.	D-Galactose	CID:6036	+	-	-	-	-	No	No	No	No	No	No	No	No
81.	4-Hydroxycinnamic acid (p-Coumaric acid)	CID:637542	+	+	-	+	-	No	No	No	No	No	No	No	No
82.	Caffeic acid	CID:689043	+	-	-	-	-	No	No	No	No	No	No	No	No
83.	Ursolic acid	CID:64945	+	+	-	+	-	No	No	No	No	No	No	Yes	No
84.	D-Xylose	CID:135191	+	-	-	-	-	No	No	No	No	No	No	No	No
85.	L-Rhamnose	CID:25310	+	-	-	-	-	No	No	No	No	No	No	No	No
86.	D-Mannose	CID:18950	+	-	-	-	-	No	No	No	No	No	No	No	No
87.	trans-Linalool oxide (2-Furanmethanol, 5-ethenyltetrahydro-alpha,alpha,5-trimethyl-, trans-)	CID:6432254	+	+	-	-	-	No	No	No	No	No	No	No	No
88.	Limonene	CID:22311	+	+	+	+	-	No	No	No	No	No	No	No	No
89.	D-Glucose	CID:5793	+	-	-	-	-	No	No	No	No	No	No	No	No
90.	beta-Caryophyllene	CID:5281515	+	+	+	+	-	No	No	No	No	No	No	No	No
91.	2-Furanmethanol, 5-ethenyltetrahydro-alpha,alpha,5-trimethyl-, cis- (Linalool oxide B)	CID:11116492	+	+	-	+	-	No	No	No	No	No	No	No	No
92.	5-Ethyl-2(5H)-furanone	CID:16997	+	+	-	+	-	No	No	No	No	No	No	No	No
93.	Pentadecanoic acid	CID:13849	+	+	-	+	-	No	No	No	No	No	No	No	No
94.	2,6-Dimethylnaphthalene	CID:11387	+	+	+	+	-	Yes	Yes	No	Yes	No	No	No	No
95.	2-Acetylpyrrole	CID:14079	+	+	-	+	-	No	No	No	No	No	Yes	No	No
96.	Geranylacetone	CID:1549778	+	+	+	+	-	No	No	No	No	No	No	No	No
97.	Isovaleric acid	CID:10430	+	+	-	-	-	No	No	No	No	No	No	No	No
98.	Myristic acid	CID:11005	+	+	-	+	-	No	No	No	No	No	No	No	No
99.	3-Methyl-2-buten-1-OL (Prenol)	CID:11173	+	+	-	+	-	No	No	No	No	No	No	No	No
100.	2-Methoxy-4-vinylphenol	CID:332	+	+	-	+	-	Yes	No	No	No	No	Yes	No	No
101.	Acetoin	CID:179	+	+	-	+	-	No	No	No	No	No	No	No	No
102.	Octanoic acid	CID:379	+	+	-	+	-	No	No	No	No	No	No	No	No
103.	Decanoic acid (Capric Acid)	CID:2969	+	+	-	+	-	No	No	No	No	No	No	No	No
104.	Acetylpyrazine	CID:30914	+	+	-	+	-	No	No	No	No	No	No	No	No
105.	2,3-Butanediol	CID:262	+	+	-	-	-	No	No	No	No	No	No	No	No
106.	3-(Methylthio)propionaldehyde (Methional)	CID:18635	+	+	-	+	-	No	No	No	No	No	No	No	No
107.	Stearic acid	CID:5281	+	+	-	+	-	Yes	No	No	No	No	No	No	No
108.	Methyl linoleate	CID:5284421	+	+	+	+	-	Yes	No	No	No	No	No	No	No
109.	2-Heptanone	CID:8051	+	+	+	+	-	No	No	No	No	No	No	No	No
110.	2-Nonenal	CID:5283335	+	+	+	+	-	No	No	No	No	No	No	No	No
111.	2,4-Decadienal	CID:5283349	+	+	+	+	-	No	No	No	No	No	No	No	No
112.	2-Isobutyl-3-methoxypyrazine	CID:32594	+	+	+	+	-	Yes	No	No	No	No	No	Yes	No
113.	Eugenol	CID:3314	+	+	+	+	-	Yes	No	No	No	No	Yes	No	NO
114.	Nonanal	CID:31289	+	+	+	+	-	No	No	No	No	No	No	No	No
115.	Isoamyl alcohol	CID:31260	+	+	-	+	-	No	No	No	No	No	No	No	No
116.	Tiglic aldehyde	CID:5321950	+	+	-	+	-	No	No	No	No	No	No	No	No
117.	1-Octen-3-OL	CID:18827	+	+	+	+	-	No	No	No	No	No	No	No	No
118.	2-Pentylfuran	CID:19602	+	+	+	+	-	No	No	No	No	No	No	No	No

119.	Trigonelline	CID:5570	+	+	-	+	-	No	No	No	No	No	No	No	No
120.	Methyl oleate	CID:5364509	+	+	+	+	-	Yes	No	No	No	No	No	No	No
121.	2-Phenylethanol	CID:6054	+	+	-	-	-	Yes	No	No	No	No	No	No	No
122.	Cholesterol	CID:5997	+	+	+	+	-	No	No	No	No	No	No	No	No
123.	2-Methyl-1-butanol	CID:8723	+	+	-	+	-	No	No	No	No	No	No	No	No
124.	1,3-Butanediol (Butylene Glycol)	CID:7896	+	+	-	-	-	No	No	No	No	No	No	No	No
125.	Methyl palmitate	CID:8181	+	+	+	+	-	Yes	No	No	No	No	No	No	No
126.	Indole	CID:798	+	+	+	+	-	Yes	No	No	No	No	No	No	No
127.	Furfuryl alcohol	CID:7361	+	+	+	+	-	No	No	No	No	No	No	No	No
128.	Furfural	CID:7362	+	+	-	+	-	No	No	No	No	No	Yes	No	No
129.	2-Methylbutanoic acid	CID:8314	+	+	-	+	-	No	No	No	No	No	No	No	No
130.	Maltol	CID:8369	+	+	-	+	-	No	No	No	No	No	No	No	No
131.	2,3-Butanedione	CID:650	+	+	-	+	-	No	No	No	No	No	Yes	No	No
132.	Docosanoic acid (Behenic Acid)	CID:8215	+	+	-	-	+	Yes	No	No	No	No	No	Yes	No
133.	Methyl stearate	CID:8201	+	+	+	+	-	Yes	No	No	No	No	No	No	No
134.	Pentanal (Valeraldehyde)	CID:8063	+	+	-	+	-	No	No	No	No	No	No	No	No
135.	Palmitic acid	CID:985	+	+	-	+	-	No	No	No	No	No	No	No	No
136.	Hexanoic acid (Caproic Acid)	CID:8892	+	+	-	+	-	No	No	No	No	No	No	No	No
137.	Pyrazine	CID:9261	+	+	-	+	-	No	No	No	No	No	No	No	No
138.	1-Pentanol	CID:6276	+	+	-	+	-	No	No	No	No	No	No	No	No
139.	4-Vinylphenol	CID:62453	+	+	+	+	-	Yes	No	No	No	No	No	No	No
140.	Vitamin E	CID:14985	+	+	+	-	-	No	No	No	Yes	No	No	No	No
141.	gamma-Butyrolactone	CID:7302	+	+	-	+	-	No	No	No	No	No	No	No	No
142.	Oleic acid	CID:445639	+	+	-	+	-	Yes	No	No	No	No	No	No	No
143.	Linalool	CID:6549	+	+	+	+	-	No	No	No	No	No	No	No	No
144.	Nonanoic acid	CID:8158	+	+	-	+	-	No	No	No	No	No	No	No	No
145.	beta-Pinene	CID:14896	+	+	+	+	-	No	No	No	No	No	No	No	No
146.	2,3-Hexanedione	CID:19707	+	+	-	+	-	No	No	No	No	No	No	No	No
147.	ar-Turmerone	CID:160512	+	+	+	+	-	Yes	No	No	No	No	No	No	No
148.	Campesterol	CID:173183	+	+	+	+	-	No	No	No	No	No	No	No	No
149.	Kahweofuran	CID:526931	+	+	+	+	-	Yes	No	No	No	No	No	No	No
150.	Methyl isovalerate	CID:11160	+	+	-	+	-	No	No	No	No	No	No	No	No
151.	Naphthalene	CID:931	+	+	+	+	-	Yes	No	No	No	No	No	No	No
152.	Benzaldehyde	CID:240	+	+	-	+	-	Yes	No	No	No	No	No	No	No
153.	Ethylbenzene	CID:7500	+	+	+	+	-	Yes	No	No	No	No	No	No	No
154.	Toluene	CID:1140	+	+	+	+	-	No	No	No	No	No	No	No	No
155.	3-Carene	CID:26049	+	+	+	+	-	No	No	No	No	No	No	No	No
156.	Arachidic acid	CID:10467	+	+	-	+	-	Yes	No	No	No	No	No	Yes	No
157.	Guaiacol	CID:460	+	+	-	+	-	No	No	No	No	No	No	No	No
158.	Ethyl salicylate	CID:8365	+	+	+	+	-	Yes	No	No	No	No	Yes	No	No
159.	cis-Isoeugenol	CID:1549041	+	+	+	+	-	Yes	No	No	No	No	No	Yes	No
160.	3-Ethyl-2-hydroxy-2-cyclopenten-1-one	CID:62752	+	+	+	+	-	No	No	No	No	No	No	No	No
161.	2-Acetyl-1-methylpyrrole	CID:61240	+	+	-	+	-	No	No	No	No	No	Yes	No	No
162.	3-Methylcyclopentane-1,2-dione	CID:61209	+	+	-	+	-	No	No	No	No	No	No	No	No

163.	3-Methyl-2-butenal (Senecialdehyde)	CID:61020	+	+	-	+	-	No	No	No	No	No	No	No	No
164.	2,6-Dimethylpyridine	CID:7937	+	+	-	+	-	No	No	No	No	No	No	No	No
165.	beta-Butyrolactone	CID:18303	+	+	-	+	-	No	No	No	No	No	Yes	No	No
166.	Limonene	CID:22311	+	+	+	+	-	No	No	No	No	No	No	No	No
167.	trans-Linalool oxide	CID:6432254	+	+	-	+	-	No	No	No	No	No	No	No	No
168.	xi-3,5-Dimethyl-2(5H)-furanone	CID:318158	+	+	-	+	-	No	No	No	No	No	No	No	No
169.	2-Ethyl-3-methylpyrazine	CID:27457	+	+	-	+	-	No	No	No	No	No	No	No	No
170.	2,2,4,6,6-Pentamethylheptane	CID:26058	+	+	+	+	-	Yes	No	No	No	No	No	No	No
171.	3-Ethyl-2,5-dimethylpyrazine	CID:25916	+	+	-	+	-	No	No	No	No	No	No	No	No
172.	Dimethyl sulfoxide	CID:679	+	+	-	+	-	No	No	No	No	No	No	No	No
173.	1H-pyrrole	CID:12668434	+	+	-	+	-	No	No	No	No	No	No	No	No
174.	gamma-Valerolactone	CID:7921	+	+	-	+	-	No	No	No	No	No	No	No	No
175.	Furan, 2,2'-methylenebis-	CID:7092	+	+	-	+	-	Yes	No	No	No	No	No	No	No
176.	2-(Methoxymethyl)furan (Furfuryl methyl ether)	CID:61661	+	+	-	+	-	No	No	No	No	No	No	No	No
177.	1H-Pyrrole, 1-pentyl-	CID:557117	+	+	+	+	-	No	No	No	No	No	No	No	No
178.	Furfuryl methyl sulfide	CID:518937	+	+	-	+	-	No	No	No	No	No	No	No	No
179.	2-Acetyl-3-methylpyrazine	CID:32093	+	+	-	+	-	No	No	No	No	No	No	No	No
180.	Furfuryl formate	CID:556916	+	+	-	+	-	No	No	No	No	No	No	No	No
181.	Nerol	CID:643820	+	+	+	+	-	No	No	No	No	No	No	No	No
182.	Linalool oxide B	CID:11116492	+	+	-	+	-	No	No	No	No	No	No	No	No
183.	Atractyligenin	CID:11045436	+	-	-	+	-	No	No	No	No	No	No	Yes	No
184.	2-Butylfuran	CID:20534	+	+	+	+	-	No	No	No	No	No	No	No	No
185.	beta-Sitosterol	CID:222284	+	+	+	+	+	No	No	No	No	No	No	No	No
186.	Stigmasterol	CID:5280794	+	+	+	+	+	No	No	No	No	No	No	No	No
187.	Geraniol	CID:637566	+	+	+	+	-	No	No	No	No	No	No	No	No
188.	2-Propylpyrazine	CID:87466	+	+	-	+	-	No	No	No	No	No	No	No	No
189.	o-Tolualdehyde O-pentafluorophenylmethyl-oxime	CID:91727711	+	+	-	+	+	Yes	Yes	No	Yes	No	No	Yes	No
190.	Cafestol	CID:108052	+	+	-	+	+	Yes	No	No	No	No	No	No	No
191.	Kahweol	CID:114778	+	+	-	+	+	No	No	No	Yes	No	No	No	No
192.	Ambap17754-44-8	CID:118701294	-	-	-	-	-	No	No	No	No	No	No	No	No
193.	[(2S,3R,4S,5S,6R)-3,4,5-trihydroxy-6-(hydroxymethyl)oxan-2-yl] (1S,4S,5R,9R,10S,13R,14R)-10,14-dihydroxy-14-(hydroxymethyl)-5,9-dimethyltetracyclo[11.2.1.01,10.04,9]hexadecane-5-carboxylate	CID:101603219	-	-	-	-	-	No	No	No	No	No	No	No	No
194.	4-Ethylphenol	CID:31242	+	+	+	+	-	Yes	No	No	No	No	No	No	No
195.	4-Ethyl-2-methoxyphenol	CID:62465	+	+	+	+	-	No	No	No	No	No	No	Yes	No
196.	Nicotinic acid	CID:938	+	+	-	+	-	No	No	No	No	No	No	No	No
197.	16-O-Methylcafestol	CID:68103163	+	+	-	+	+	Yes	Yes	No	Yes	No	No	No	No
198.	2-Ethylphenol	CID:6997	+	+	+	+	-	Yes	No	No	No	No	No	No	No
199.	Theobromine	CID:5429	+	+	-	+	-	No	No	No	No	No	Yes	Yes	No
200.	Trigonelline	CID:5570	+	+	-	+	-	No	No	No	No	No	No	No	No

201.	Allantoic acid	CID:203	+	-	-	-	-	No	No	No	No	No	No	No	No
202.	Allantoin	CID:204	+	-	-	-	-	No	No	No	No	No	No	No	No
203.	L-(+)-Arabinose	CID:5460291	+	-	-	-	-	No	No	No	No	No	No	No	No
204.	3,4-Dicaffeoylquinic acid	CID:5281780	-	-	-	-	-	No	No	No	No	No	No	No	No
205.	4,5-Dicaffeoyl quinic acid	CID:13887346	-	-	-	-	-	No	No	No	No	No	No	No	No
206.	Stigmast-7-enol	CID:3080632	+	+	+	+	+	No	No	No	No	No	No	No	No
207.	Caffeic acid	CID:689043	+	-	-	-	-	No	No	No	No	No	No	No	No
208.	Fucosterol	CID:5281326	+	+	+	+	+	No	No	No	No	No	No	No	No
209.	24-Methylenecycloartanol	CID:94204	+	+	+	+	-	No	No	No	No	No	No	No	No
210.	2,3,5-Trimethylphenol	CID:12769	+	+	+	+	-	No	No	No	No	No	No	No	No
211.	Vitamin E	CID:14985	+	+	+	-	-	No	No	No	Yes	No	No	No	No
212.	Cyclohexanecarboxylic acid, 3-[[3-(3,4-dihydroxyphenyl)-1-oxo-2-propenyl]oxy]-1,4,5-trihydroxy-, (1S,3R,4S,5R)- (Isochlorogenic acid)	CID:73081	-	-	-	-	-	No	No	No	No	No	No	No	No
213.	Melatonin	CID:896	+	+	-	+	-	Yes	No	No	No	No	No	No	No
214.	Serotonin	CID:5202	+	-	-	+	-	Yes	No	No	No	No	No	No	No
215.	Fontanesine B	CID:132524734	+	+	+	+	+	Yes	No	No	Yes	Yes	No	Yes	No
216.	beta-Carboline (Norhaman)	CID:64961	+	+	-	+	-	Yes	No	No	No	No	Yes	No	No
217.	Vasicine	CID:442929	+	+	-	-	-	No	No	No	No	No	Yes	No	No
218.	Corosolic acid	CID:6918774	+	-	-	+	-	No	No	No	No	No	No	Yes	No
219.	Crocetin	CID:5281232	+	-	-	+	-	No	No	No	No	No	No	No	No
220.	3,4-Dihydroxybenzoic acid	CID:72	+	-	-	-	-	No	No	No	No	No	No	No	No
221.	2-Hydroxybenzoic acid	CID:338	+	+	-	-	-	No	No	No	No	No	No	No	No
222.	Syringic acid	CID:10742	+	-	-	-	-	No	No	No	No	No	No	No	No
223.	3-Methylbenzoic acid	CID:7418	+	+	+	+	-	No	No	No	No	No	No	No	No
224.	4-Hydroxybenzoic acid 4-O-glucoside	CID:440186	+	-	-	-	-	No	No	No	No	No	No	No	No
225.	4-O-beta-D-glucosyl-4-coumaric acid	CID:9840292	+	-	-	-	-	No	No	No	No	No	No	No	No
226.	Cinnamic acid	CID:444539	+	+	+	+	-	No	No	No	No	No	No	No	No
227.	3′-O-Methyl-(−)-epicatechin-7-O-glucuronide	CID:101190386	+	-	-	-	-	No	No	No	No	No	No	No	No
228.	Rosmarinic acid	CID:5281792	+	-	-	-	-	No	No	No	No	No	No	No	No
229.	Epicatechin	CID:72276	+	-	-	-	-	No	No	No	No	No	No	No	No
230.	Piceatannol	CID:667639	+	-	-	-	-	Yes	No	Yes	Yes	No	No	No	No
231.	Hydroxytyrosol	CID:82755	+	+	-	-	-	No	No	No	No	No	Yes	No	No
232.	Carnosol	CID:442009	+	-	-	+	-	No	No	No	Yes	No	No	No	No
233.	Carnosic acid	CID:65126	+	-	-	+	-	No	No	No	No	No	No	No	No
234.	Pyrogallol	CID:1057	+	-	-	-	-	No	No	No	No	No	No	No	No
235.	Octanal	CID:454	+	+	+	+	-	No	No	No	No	No	No	No	No
236.	Furfural	CID:7362	+	+	-	+	-	No	No	No	No	No	Yes	No	No
237.	6-Methyl-5-hepten-2-one	CID:9862	+	+	+	+	-	No	No	No	No	No	No	No	No
238.	Damascenone	CID:5366074	+	+	+	+	-	No	No	No	No	No	No	No	No
239.	5,5-dimethyl-4-propan-2-ylidene-1H-pyrazole	CID:557693	+	+	-	+	-	No	No	No	No	No	No	No	No
240.	1-Octadecanesulphonyl chloride	CID:66281	+	+	-	-	-	Yes	No	No	No	No	No	No	No

BBB: Blood Brain Barrier; HIA: Human Intestinal Absorption; P-gp: P-glycoprotein inhibition; hERG: Human ether a-go-go gene inhibition; AT: Ames Toxicity; HT: Hepatoxicity Test.
