# Supplementary figures and images for: Pharmacological assessment of Coffea arabica compounds as potential therapeutics for cervical cancer
Source: Bioinform Adv. 2025 Jun 5;5(1):vbaf132. doi: 10.1093/bioadv/vbaf132 (PMC12212767; doi:10.1093/bioadv/vbaf132)

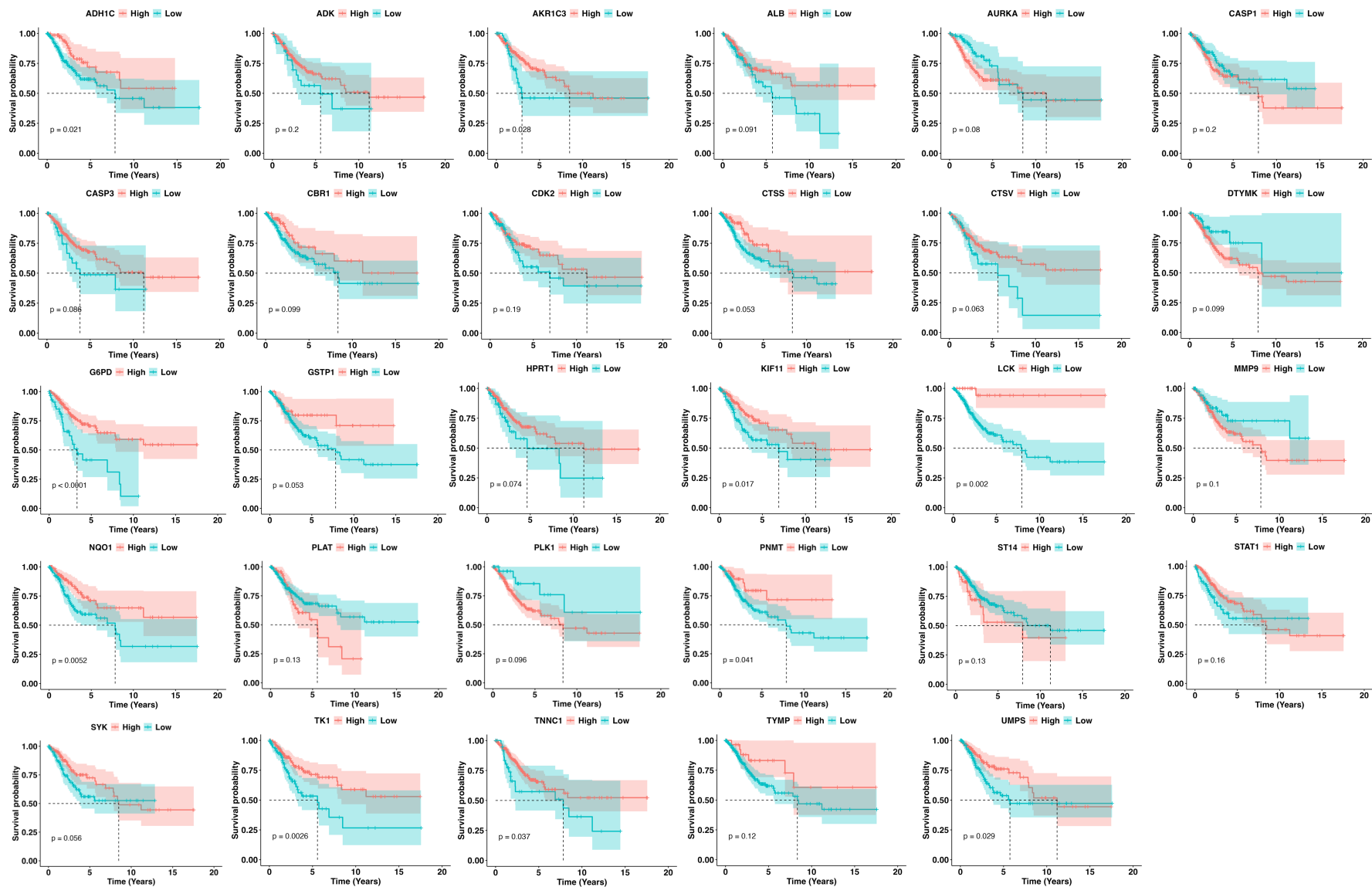

Supplement: vbaf132_Supplementary_Data [file vbaf132_supplementary_data.zip › CC_C_arabica_Manuscript_Supp_Figure_1.pdf]
